# The Relation of Fat to Nervous Disease1Read at the 36th annual meeting of the Medical Association of Central New York, held at Auburn, September 22, 1903.

**Published:** 1903-12

**Authors:** Bradford C. Loveland

**Affiliations:** Syracuse, N. Y.


### BUFFALO MEDICAL JOURNAL.

VOL. XLIII.—LIX. DECEMBER, 1903. No. 5.

### ORIGINAL COMMUNICATIONS.

### The Relation of Fat to Nervous Disease.[1]

By BRADFORD C. LOVELAND, M. D., Syracuse, N. Y.

THIS paper will present briefly the results of clinical observation, and it is a matter of regret that the resources of the laboratory have not been available in confirmation of all the statements made. Fat exists to some degree in all living animals, and its proportionate amount is in some unknown way under the influence of the nervous system. The trophic function of the nervous system governs also the growth and development of muscles and other tissues as well as fat. It is a common clinical observation to note the rapid loss of flesh in melancholia, the impossibility of getting flesh on a case of anorexia nervosa, and like observations where the cause behind the disordered function was a diseased mental state.

It is not so common to note rapid and extreme increase in fat following an unusually distressing bereavement, or after hemiplegia. I have a picture of a woman who when it was taken weighed 213 lbs. She was 5 feet 1 inch in height and weighed, when married, at the age of 22, 90 lbs. Her weight gradually increased until at 45 years she weighed 140 lbs. Her weight remained about the same until she was about 58 years old, when she was called to a distant state to attend the funeral of a brother who died suddenly and unexpectedly. On her return she found that her husband had died during her absence, equally unexpectedly. From this time she rapidly increased in flesh till she reached 213 lbs., and with her small bones it was a painful burden to carry.

1. Read at the 36th annual meeting of the Medical Association of Central New York, held at Auburn, September 22, 1903.

Another woman, after an attack of hemiplegia which left her mind weakened as well as her body, steadily increased in flesh until it took two nurses and a tackle to move her from her chair to her bed, and while I do not know her exact weight, I think it was more than twice what it had been in health, or nearly 300 lbs. We know that in the early stages of paresis the patient frequently accumulates considerable flesh, while in acute mania, and acute melancholia, there is a remarkably rapid loss of flesh. I knew two people both, strangely enough, having been victims of a runaway accident, resulting in a severe nervous shock, who afterwards developed that rather rare disorder called adiposus dolorosa, or Dercum's disease, one of which was reported in the American Textbook of Nervous Diseases. It seems strange that such remarkable and widely divergent results as these referred to should be brought about through the influence of the nervous system, but they are sufficient to show the very important relation that exists between the nervous system and the production of fat.

Fat is the stored up heat producing fuel of the body, in other words, that portion of the hydrocarbon elements of our food which has been absorbed and not burned up, or oxidised. These carbonhydrates may go through the body undigested, and therefore are incapable of absorption; may be oxidised in the body; partly oxidised in the body; or transformed into fat and stored in the tissues. Starchy indigestion may be a nervous affair, or an organic difficulty, and careful study will decide which. Oxidation or combustion within the body is the natural result of most of the absorbed carbonaceous foods.

Imperfect oxidation may be the result of over ingestion of carbonaceous foods, insufficient exercise, or a depressed state of the mind and nervous system, and in its turn may cause many a nervous irritation, and even mental depression. I have seen at least one patient in whose case I could tell whether or not he had eaten sugar at a meal by the depth of his depression three or four hours afterward. The sugar suspended, his depression lifted. Fat is the result in healthy persons, of a very small proportion of our carbonaceous foods, and its function is largely as a cushion and protection to nerves and other delicate structures, and as a reserve supply to keep up the bodily heat. Yet excess of fat seems to impede oxidation, lessen the bodily warmth, and in some instances to add more burden to already over-taxed nerves.

I was called in consultation recently in a neighboring town, where I found a woman 5 feet 3 inches high, weighing about 200 pounds lying in bed, where she had been for three months or more, suffering with what is called neurasthenia, but in this case really hysteria. She showed unquestionable evidence of uric acid excess, both in the physical signs and in the urine. Her worst complaint was of a trembling and giving out in her legs and hips on any exertion. She had been taking frequent feeding, and used a considerable quantity of milk and eggs in her diet. It was evident that she did not need more flesh, but more oxidation, and elimination, and a marked change in her diet was advised. This case naturally suggests fat as a guide to diagnosis and prognosis, and as an index of improvement in the functional neuroses, or psychoses.

Given two persons suffering with much the same neurasthenic symptoms, the one fat, possibly too fat, the other lean, and it will usually be found that the hysterical element is much stronger in the fat than in the lean one, and a prognosis of recovery can be given with more of hope in the thin one than in the fat one. This is especially true if the thin patient has at one time been fleshy, and the digestive organs will stand forced feeding. Nourishment will answer for the watchword in the thin case, but we will have to choose some other motto for the fat one, say "elimination and oxidation," or "mental discipline, and increased mental control," or some combination of these.

Of fat as an index of improvement it may be said that in a thin person suffering from a neurosis or psychosis an increase in flesh is always a sign of improved nutrition, especially if blood conditions improve correspondingly, and hence encouraging to both patient and physician. But if a patient has been long in the toils of such an affliction it brings in another element to contend with, which is the question of habit, and that lack of self-confidence which so often seems to enslave the nervous invalid, and we cannot reasonably expect the strength and self-control to keep quite pace with the gain in flesh. The increase in flesh, however, may be used as a very efficient lever through which to apply mental therapeutics, which this class of patients generally need.

The trophic peculiarities of different individuals vary widely, and as we have all observed some people will keep fat on an amount and variety of food that will not keep others fat, but careful observation and definite prescribing of diet for individual cases and noting result, will soon enable one to prescribe diet for any given case so accurately that he can foretell whether they will gain or lose, and at what rate. One cannot, of course, be absolutely correct in his prognosis in this regard any more than in many other conditions, but he can be correct often enough to make it very encouraging to his patients and satisfactory to himself. The class of patients best suited to this sort of prescribing are the so-called nervous dyspeptics, or gastric neurasthenics, who have become thin and anxious, leaving off one article of food after another thinking it hurt them, till they are really almost starved.

I have seen these sufferers after "doctoring" with medicines and digestives for years, and having dwindled to a mere shadow of their former selves, put on a diet, prescribed with the same care that we would prescribe quinine in malarial fever, gain from one to five pounds a week steadily for months till their recovery was complete.

The points it is desired to bring out in this paper are first, that the depositing of fat in the body is under the functional control of the nervous system; second, that a careful study of the patient with regard to the question of fat may be of great help both in diagnosis and prognosis; third, that a physician may so know his patient that he can prescribe a diet and tell the patient with a reasonable degree of certainty, whether he will lose, or keep the same, or gain flesh, and at what rate; fourth, that an increase in flesh may be taken as an index of improvement in acute mania, in melancholia, and in the thin neurasthenic, as well as in the tuberculous, where it has long been regarded as such.

FAYETTE PARK.

